# High-throughput electrophysiological assays for voltage gated ion channels using SyncroPatch 768PE

**DOI:** 10.1371/journal.pone.0180154

**Published:** 2017-07-06

**Authors:** Tianbo Li, Gang Lu, Eugene Y. Chiang, Tania Chernov-Rogan, Jane L. Grogan, Jun Chen

**Affiliations:** 1 Department of Biochemical and Cellular Pharmacology, Genentech Inc., South San Francisco, California, United States of America; 2 Department of Cancer Immunology, Genentech Inc., South San Francisco, California, United States of America; Universita degli Studi di Napoli Federico II, ITALY

## Abstract

Ion channels regulate a variety of physiological processes and represent an important class of drug target. Among the many methods of studying ion channel function, patch clamp electrophysiology is considered the gold standard by providing the ultimate precision and flexibility. However, its utility in ion channel drug discovery is impeded by low throughput. Additionally, characterization of endogenous ion channels in primary cells remains technical challenging. In recent years, many automated patch clamp (APC) platforms have been developed to overcome these challenges, albeit with varying throughput, data quality and success rate. In this study, we utilized SyncroPatch 768PE, one of the latest generation APC platforms which conducts parallel recording from two-384 modules with giga-seal data quality, to push these 2 boundaries. By optimizing various cell patching parameters and a two-step voltage protocol, we developed a high throughput APC assay for the voltage-gated sodium channel Nav1.7. By testing a group of Nav1.7 reference compounds’ IC_50_, this assay was proved to be highly consistent with manual patch clamp (R > 0.9). In a pilot screening of 10,000 compounds, the success rate, defined by > 500 MΩ seal resistance and >500 pA peak current, was 79%. The assay was robust with daily throughput ~ 6,000 data points and Z’ factor 0.72. Using the same platform, we also successfully recorded endogenous voltage-gated potassium channel Kv1.3 in primary T cells. Together, our data suggest that SyncroPatch 768PE provides a powerful platform for ion channel research and drug discovery.

## Introduction

Ion channels are involved in a broad spectrum of physiological processes such as neuronal firing, muscle contraction, hormone secretion and T cell activation [1]. Many ion channels have been identified as important therapeutic targets, including the voltage-gated sodium channel Nav1.7 and the voltage gated potassium channel Kv1.3. Nav1.7 is preferentially expressed in sensory neurons and is implicated as the threshold channel for pain sensation [2,3]. In humans, loss of function mutations of Nav1.7 lead to congenital insensitivity to pain (CIP), whereas gain of function mutations of Nav1.7 cause inherited erythermalgia (IEM) and proxysmal extreme pain disorder (PEPD) syndromes [4–6], therefore Nav1.7 antagonists should have an application in pain management. Kv1.3 regulates membrane potential and Ca^2+^ signaling in T cells, and its expression is enhanced in CD4^+^ and CD8^+^ cells following T cell receptor activation [7–9]. The inhibition of Kv1.3 suppresses Ca^2+^-signaling, cytokine production, and proliferation of autoantigen-specific T cells. Therefore Kv1.3 blockers may have utility in autoimmune diseases treatment [10]. Despite the recent advances in channelopathies and protein structures, the discovery of ion channel therapeutics is still facing a major challenge from the limitation of assay technologies.

Several radioisotope and fluorescent dye based ion channel assay technologies, such as ligand binding, ion flux and membrane potential assays, have existed for several decades. They are cost efficient and amenable to high throughput screening. However, these assays have significant limitations. For instance, the ligand binding assays measure competitive binding and cannot elucidate the mechanism of a compound’s action (e.g., agonism vs. antagonism). Ion flux and membrane potential assays reply on non-physiological stimuli and can only measure channel function indirectly, via radiation, atomic absorption or fluorescent signals. These assays are also prone to artifacts due to auto-fluorescence, ionophore and cellular toxicity [11–14]. In contrast, patch clamp electrophysiology directly measures ionic current, and precisely control membrane voltage, therefore allowing functional measurements at different states of the channels (e.g., open, closed, inactivated states). Although electrophysiology is considered as the gold standard method for ion channel study, it is extremely labor intensive and low throughput. On average, an experienced electrophysiologist can generate about 20 data points a day, whereas a typical chemical library consists of millions of compounds. Besides drug screening, the challenges also exist for characterizing ion channels in native or primary cells. For example, it is difficult to study endogenous ion channels in primary T cells due to their small size and fragile membrane.

To overcome these challenges, many automated patch clamp (APC) technology platforms have been developed in the past decade [15]. These include lipid bilayer recording (e.g., Orbit, from Nanion Technologies), *Xenopus* Oocytes two-electrode recording (e.g., OpusXpress, from Molecular Devices), glass pipette electrode recording (e.g., FlyScreen 8500, from Flyion), and most notably, chip or plate based planar recording technologies (IonWorksHT, Quattro, PatchXpress 7000A, IonWorks Quattro and Barracuda from Molecular Devices; QPatch16, QPatch HT, Qube from Sophion; Port-a-Patch, Patchliner SyncroPatch 96, 384PE, 768PE from Nanion). Among the planar platforms, IonWorks Barracuda, Qube and SyncroPatch 384PE/768PE have gained the most attention due to their high throughput, i.e., recording 384 or more cells in parallel.

IonWorks Barracuda was launched in 2010 and was applied for compound screening on hERG, Ca_V_2.2 and Nav channels [16–18]. Barracuda uses perforated patch configuration, the seal resistance was ~120 MΩ for single-hole mode and ~35 MΩ for population patch mode (64 holes). In 2014, Qube (Biolin Scientific, Sweden) and SyncroPatch (Nanion Technologies, German), were introduced with promised giga-seal data quality. Both platforms use 384 channels digital amplifier and 384 pipetting robot, borosilicate glass based single- or multi-hole chips, and programmable negative pressure to achieve whole cell configuration. However, they also differ in many regards. For example, Qube adopts an in chip micro-fluid design to enable solution exchange; while SyncroPatch uses a liquid handler (e.g., Biomek FX) so the system can be integrated for automation. SyncroPatch also can integrate two 384 modules into one robot platform, so 768 well parallel recording is feasible.

In this study, we implemented SyncroPatch 768PE, the currently highest throughput APC platform, by testing different voltage-gated ion channels, including Nav1.1 to 1.7 and Kv1.3. We optimized various parameters to ensure quality control and improve success rate. We benchmarked reference compounds and conducted a pilot screen of 10,000 compounds against Nav1.7. We also successfully recorded endogenous Kv1.3 currents in rat immune T cells.

## Materials and methods

### Cell line construction and stable expression

Human Nav1.7 (SCN9A) cDNA was cloned into a mammalian expression vector for constitutive expression. DNA was prepared with HiSpeed Plasmid Kit (Qiagen, Chatsworth, CA) and was transfected into chinese hamster ovary (CHO)-S15 cell, by using Lipofectamine 2000 (Thermofisher, Waltham, MA). Pool of transfected cell was cultured with selection antibiotics for 1 week then re-seeded in 384 well plates for single clone isolation. 48 individual clones were selected for expansion and were tested by manual patch clamp and SyncroPatch768PE. The highest expression stable single clone was chosen and named as CHO-Nav1.7. Similarly, constitutive expression human Nav1.1 (SCN1A), Nav1.2 (SCN2A), Nav1.5 (SCN5A), Nav1.6 (SCN8A) and Kv1.3 (KCNA3) cell lines were generated using either Chinese hamster lung (CHL) or CHO host cells. Human Nav1.3 (SCN3A) and Nav1.4 (SCN4A) expression CHO cell lines were purchased from ChanTest (Cleveland, OH), and Neuroblastoma cell line NG108-15 was purchased from Sigma-Aldrich (St. Louis, MO).

For cell culture, CHO cells were maintained in Ham’s F-12 media supplemented with 10% fetal bovine serum (Clontech, Mountain View, CA), 100 U/mL of penicillin G sodium, 100 μg/mL of streptomycin sulfate, and appropriate selection antibiotics. CHL cells were maintained in Dulbecco’s Modified Eagle’s Medium (DMEM) with high glucose and supplemented with 10% fetal bovine serum, 100 U/mL of penicillin G sodium, 100 μg/ mL of streptomycin sulfate, 2 mM Glutamine and the appropriate selection antibiotics. Neuroblastoma cells (NG108-15) were maintained in DMEM high glucose medium supplemented with 10% fetal bovine serum, 100 U/mL of penicillin G sodium, 100 μg/ mL of streptomycin sulfate and 2 mM Glutamine. All cells were cultured at a humidified 5% CO_2_ incubator at 37°C to 60% confluent, and then changed to 32°C 1 day before recording. Cell density was 80% confluent at the time of harvest. Cells were harvested by washing twice with 20 mL Hank’s Balanced Salt Solution (HBSS) without Ca^2+^ and Mg^2+^ and treatment with 4 mL of accutase (Innovative Cell Technologies, San Diego, CA) solution for 2 minutes. Cells were transferred to a 50-mL conical tube with addition of 30 mL Hank's Balanced Salt Solution (HBSS) and pipette 5 times to break up cell clumps. After centrifuging and re-suspension, cells were prepared at a density of 2x10^5^ cell/mL in CHO-S-SFM II medium (ThermoFisher, MA).

### Rat T cell preparation

*Kcna3*^-/-^ (Kv1.3 knockout, previously described in ref. 8) and WT Dark Agouti rats (Charles River Labs) were housed and maintained at Genentech in accordance with American Association of Laboratory Animal Care guidelines. 8–10 wk old female rats were used in all experiments. All experimental animal studies were conducted under the approval of the Institutional Animal Care and Use Committees of Genentech Lab Animal Research.

Primary T cells were harvested from spleens from animals that were euthanized by isoflurane inhalation to effect. CD4^+^ T cells were prepared by using ACK lysis buffer to remove red blood cell and an EasySep Rat CD4^+^ T Cell Isolation Kit (Stem Cell Technologies). Purity of isolated cells was validated by flow cytometry to be >95% and seeded in anti-rat CD3 (BD Biosciences, 5 μg/ml in PBS) pre-coated 6-well plate at 1 x 10^6^ cell/well with 6 ml RPMI 1640 medium supplemented with 10% FBS, 2 mM glutamine, 2 μM 2-ME, 1 mM sodium pyruvate, 100 U/ml penicillin, 100 μg/ml streptomycin and 2 μg/ml soluble anti-rat CD28 (BD Biosciences) for primary stimulation in a humidified incubator at 37°C for 3 days. Then cells were collected and dead cells were removed using Dead Cell Removal Kit (Miltenyi Biotec). Cell viability and activation rate were validated using flow cytometry for higher than 85% and 90% respectively. Cell viability and activation status (by CD25 expression) were assessed by flow cytometry to be >90% and >95% respectively.

Ethics Statement: All animals used in this study were housed and maintained at Genentech in accordance with American Association of Laboratory Animal Care guidelines. All experimental studies were conducted under protocols (16–0927 and 16-0927A) approved by the Institutional Animal Care and Use Committee of Genentech Lab Animal Research in an AAALACi-accredited facility in accordance with the Guide for the Care and Use of Laboratory Animals and applicable laws and regulations.

### Solutions and compound preparation

Tetrodotoxin (TTX) was purchased from Fisher Scientific (Waltham, MA); Kv1.3-specific channel blocker ShK was purchased from Bachem (Torrance, CA); all other chemical reagents were purchased from Sigma-Aldrich (St. Louis, MO) or made at Genentech. For Kv1.3 recordings, the intracellular solution contained (in mM): 50 KCl, 60 KF, 10 NaCl, 20 EGTA and 10 HEPES (pH 7.2, osmolarity 285 mOsm), and extracellular solution contained (in mM): 140 NaCl, 4 KCl, 2 CaCl_2_, 1 MgCl_2_, 5 Glucose and 10 HEPES (pH 7.4, osmolarity 300 mOsm). For Nav channels, the intracellular solution consisted of (in mM): 50 CsCl, 10 NaCl, 60 Cs-Fluoride, 20 EGTA, 2 Mg-ATP, 0.3 Na-GTP, and 10 HEPES (pH adjusted to 7.2 with CsOH and osmolarity adjusted to 285 mOsm); and extracellular solution contained (in mM): 140 NaCl, 4 KCl, 1 MgCl_2_, 2 CaCl_2_, 5 D-Glucose monohydrate, 10 HEPES (pH adjusted to 7.4 with NaOH and osmolarity adjusted to 300 mOsm).

Reference compounds were prepared in dimethyl sulfoxide (DMSO) at 10 mM and stored frozen. Dose response compound plates were prepared by serial diluting stock compound solutions into DMSO, and backfilling 200 nL to each well in a 384-well plate using an Echo 550 (Labcyte, CA). Compounds were then diluted 1:500 with extracellular solution using a Multidrop Dispenser (Thermo Scientific). The final DMSO content is 0.2% DMSO. Screening compounds were prepared by Genentech compound management group in predispensed 384-well compound plates. For 10,000 compounds screening, 200 nL stock compound solution per well was injected to 384-well compound plate, and working compound solution were prepared at 20 μM freshly by adding 100 μL extracellular solution within 6 hours of assays. Working compound solution was diluted 3 times in recording well by adding 20 μL to 40 μl external solution to reach 6.7 μM final concentration. In the end of each compound test, full block solution was applied to reach, TTX 2 μM for Nav1.7, Tetracaine 2 mM for Nav1.5 and ShK 1 nM for Kv1.3, as the 100% inhibition control for each ion channel target. All tests were performed with n ≥ 4.

### Conventional patch-clamp recording

Manual patch clamp recordings were conducted in the whole-cell configuration as described previously [19]. Patch pipettes were pulled from PG150T glass (Warner Instruments, CT) to tip diameter of 2–4 μm after heat polishing. To compare biophysical properties between manual and SyncroPatch, current–voltage (IV) relationship, steady-state inactivation (V½) and recovery time constants were measured. Compounds were applied using a Perfusion Fast Step device SF-77B (Warner Instruments) controlled by the data acquisition program PatchMaster v2.90. For Nav pharmacology test, a two-state protocol was developed by balancing tonic and state dependent Nav1.7 inhibitors’ sensitivity and current stability to facilitate high-throughput screening. Briefly, cell membrane holding voltage (Vm) was set at -120 mV, a 20 ms depolarizing pulse to -10 mV was used to elicited closed state current, and after holding at -40 mV for 4 s followed by a 20 ms step at -120 mV, another 20 ms depolarizing pulse to -10 mV was used to elicited inactivated state current. The protocol was run every 10 s. Under this protocol, the currents were stable and we could assess closed state and inactivated state block. For Kv1.3 channel recording, the holding potential was set at -80 mV, and currents were elicited by depolarizing voltage steps from −60 mV to +40 mV (10 mV increments) for kinetic study, or by repetitive pulses to 40 mV for pharmacological studies. The sweep interval was set at 30 s to avoid rundown. Data were collected at 50 kHz and filtered at 10 kHz using an EPC-10 amplifier (HEKA Electronic, Germany). Patch-clamp measurements are presented as the mean ± SEM.

### Automated patch-clamp recording

Automated patch-clamp recordings were performed using SyncroPatch 768PE (Nanion, München, Germany). Chips with single-hole medium resistance (5~8 MΩ) were used for recombinant cell lines and chips with high resistance (~10 MΩ) were used for primary T cell recording. Pulse generation and data collection were performed with PatchController384 V1.4.1 and DataController384 V1.3.3. Whole-cell recordings were conducted according to Nanion’s procedure. Briefly, cells were stored in a cell hotel reservoir at 10°C with shaking speed at 60 RPM. After initiating the experiment, cell catching, sealing, whole-cell formation, liquid application, recording, and data acquisition were performed sequentially. The voltage protocol consists 220 ms leak pulse to obtain seal resistance (Rseal), series resistance (Rs) and cell capacitance (Cslow), and 500 ms data processing segment. Series resistance compensation was set to 80% and currents were sampled at 10 kHz.

### Data analysis and statistics

In manual patch clamp, data was acquired using PatchMaster and analyzed with FitMaster softwares (both version 2.90, HEKA Inc.). Series resistance and capacitance were compensated automatically by the HEKA PatchMaster software. In automated patch clamp, SyncroPatch data acquisition was performed using PatchControl software (version 1.3.3, Nanion, Inc) with leak current correction model.

The voltage-dependent steady-state activation curves were assessed with the membrane potential (V_M_) held at −120 mV and a series of 20 ms test pulses ranging from −80 to +60 mV in 5 mV increments. The chord conductance (G) was calculated from peak current (I_Peak_) and reversal potential (V_Rev_) using the following equation:

$${\text{I}}_{\text{Peak}}=\text{G }\left({\text{V}}_{\text{M}}-{\text{V}}_{\text{Rev}}\right)$$

The voltage-dependent steady-state inactivation curves were assessed with a standard two-pulse protocol in which the cells were stepped from -120 mV to a preconditioning pulse ranging from -120 to 0 mV for 500 ms before a 0 mV pulse for 20 ms. Both steady-state activation curves and inactivation curves were fitted using Boltzmann function, where Y is G or I_Peak_, k is the slope factor, and V½ is Y midpoint voltage:

$$\text{Y }={\text{ Y}}_{\text{MAX}}/\left\{1\;+\text{ exp }\left[\left({\text{V}}_{\text{M}}-{\text{ V}}_{\text{½}}\right)/-\text{k}\right]\right\}$$

For compound effect analysis, compound inhibition was calculated as the percentage of peak current (I) decrease from before compound application (I_Baseline_) to the end of 10 minutes compound application (I_End_) and both being normalized to the end of experiment full block current (I_Fullblock_) using this following equation:

$$\text{Block \%}=\;\left[1-\;\frac{{\text{I}}_{\text{End}}-{\text{ I}}_{\text{Fullblock}}}{{\text{I}}_{\text{Baseline}}-{\text{I}}_{\text{Fullblock}}}\right]\times 100\text{ \%}$$

Results for each test compound concentration were calculated for mean and standard deviation (with manual patch clamp n = 4~10, APC n = 6~384) and used to generate dose-response curves.

For analysis compound IC_50_ value from manual patch clamp data, dose-response curve was calculated by fitting to four-parameter Hill equation using GraphPad Prism (version 6.05, Graphpad Software, La Jolla, CA), with constrained bottom 0 and top 1. For automated patch-clamp data, same fitting strategy was applied using SyncroPatch DataControl software (version 1.3.3, Nanion, Inc).

In 10,000 compounds screening, Z’-factor was calculated by using standard deviation (SD) and mean (M) of inhibitions from negative controls (i.e. 0.2% DMSO wells) and positive controls (i.e. full block conditions) with this following equation:

$${\text{Z}}^{\prime }\text{Factor}=1-\;\frac{\left(3\times {\text{SD}}_{\text{negative}}+3\times {\text{SD}}_{\text{positive}}\right)}{\left|{\text{M}}_{\text{negative}}-{\text{M}}_{\text{positive}}\right|}$$

Statistical significance was determined using an unpaired student’s t-test with statistical significance defined as p < 0.05 unless otherwise stated.

## Results

### Biophysical characterization of Nav1.7, Nav1.5 and Kv1.3: SyncroPatch vs. manual patch clamp

**Fig 1:**
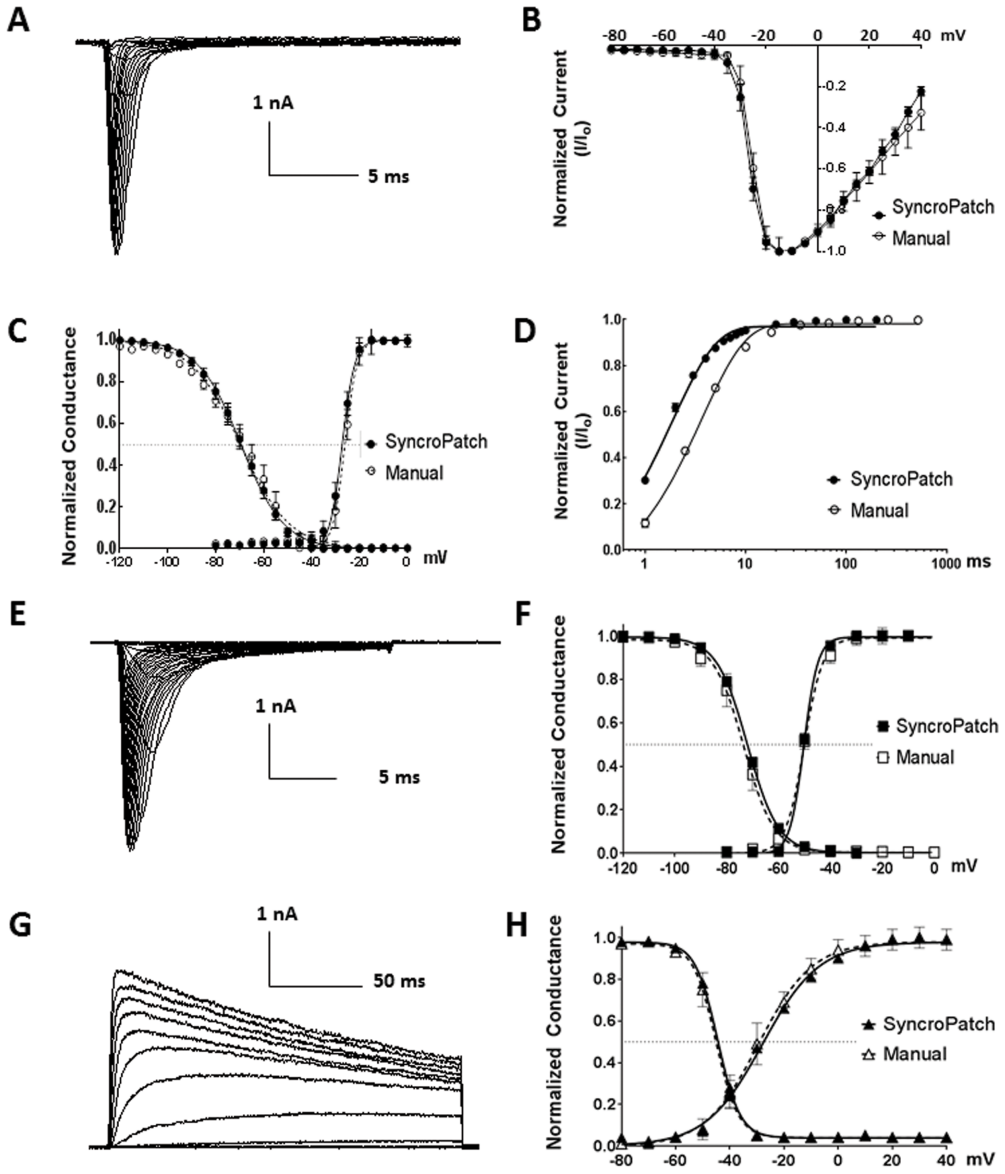
Comparison of SyncroPatch APC and manual patch clamp (MPC) by characterizing Nav1.7, Nav1.5 and Kv1.3. (A) Representative Nav1.7 current traces from APC. The currents were elicited by 20 ms test pulses (-80 to 40 mV in 5 mV increment) from a holding potential at -120 mV. (B) Overlay of Nav1.7 IV relationship curves from APC and MPC. The peaks of the IV from both systems were -10 mV. (C) Superimposition of steady-state activation and inactivation curves of Nav1.7 from APC and MPC. Inactivation currents were elicited by 20 ms test pulses at -10 mV, after 500 ms conditioning prepulses ranged from—120 to 0 mV with 5 mV increments. The smooth curves are Boltzmann fits with activation V½ and slope factors (k) from APC -27.3 ± 0.9 mV and 2.6 ± 0.2 mV/e-fold potential change; and from MPC -26.1 ± 1.6 mV and 2.4 ± 0.3 mV/e-fold potential change. For inactivation the V½ and k values are -69.4 ± 0.4 mV and 8.9 ± 0.3 mV/e-fold potential change from APC and -69.7 ± 0.7 mV and 8.3 ± 0.6 mV/e-fold potential change from MPC. (D) Peak current (elicited by -10 mV 20 ms) was plotted as a function of inter-stimulus interval (prepulse and holding Vm at -120mV) ranging from 1 ms to 1,000 ms, and fitted with one phase decay exponential equation to obtain the recovery time constant, τ = 1.85 ± 0.1 and 3.65 ± 0.2 ms from APC and MPC, respectively. (E) Representative Nav1.5 current traces from APC. (F) Superimposition of Nav1.5 steady-state activation and inactivation curves from APC and MPC. The smooth curves are Boltzmann fits with activation V½ and slope factors (k) are -50.3 ± 0.5 mV and 2.7 ± 1.0 mV/e-fold potential change from APC; and are -50.1 ± 0.4 mV and 3.5 ± 0.5 mV/e-fold potential change from MPC. For inactivation the V½ and k values are -72 ± 0.4 mV and 5.9 ± 0.4 mV/e-fold potential change from APC; and are -73.5 ± 0.7 mV and 5.8 ± 0.6 mV/e-fold potential change from MPC. (G) Representative Kv1.3 current traces from APC. (H) Superimposition of Kv1.3 steady-state activation and inactivation curves from APC and MPC. The smooth curves are Boltzmann fits with activation V½ and slope factors (k) are -27.9 ± 1.0 mV and 8.5 ± 0.4 mV/e-fold potential change from APC; and are -29.4 ± 1.6 mV and 7.9 ± 1.4 mV/e-fold potential change from MPC. For inactivation the V½ and k values are -44.5 ± 0.5 mV and 4.1 ± 0.4 mV/e-fold potential change from APC; and are -45.2 ± 0.5 mV and 4.2 ± 0.4 mV/e-fold potential change from MPC. Note that all normalized data were shown as mean ± SEM, with data points in APC n = 290 ~ 384 and MPC n = 4 ~ 10.

We first characterized biophysical properties of Nav1.7 channel using SyncroPatch APC and manual patch clamp (MPC). We derived the current–voltage (IV) relationship using a step protocol (Fig 1A). Currents obtained from SyncroPatch and MPC peaked at the same voltage of -10mV. The half-activation/inactivation voltages (V½ act./V½ inact.) and slope factors (k act./k inact.) were -27.3 ± 0.9/-69.4 ± 0.4 mV and 2.6 ± 0.2/8.9 ± 0.3 mV/e-fold potential change from APC and -26.1 ± 1.6/-69.7 ± 0.7 mV and 2.4 ± 0.3/8.3 ± 0.6 mV/e-fold potential change from MPC, with no statistically significant difference to each other (Fig 1A–1C), and also similar to literature [20]. We also derived the recovery time constants τ, which was 3.65 ± 0.2 ms from MPC, similar to a previous report [21]; and 1.85 ± 0.1 ms from APC (Fig 1D). The 1.8 ms difference in τ could be due to difference in hardwire (e.g., the highly compact 384-channel amplifier for SyncroPatch vs. standalone HEKA EPC10 amplifier for conventional MPC). Based on these results, we set the minimum time segment to 20 ms (> 10 times to the system time lag 1.8 ms) in all voltage protocols for rest of the study.

Nav1.5 is the cardiac isoform of sodium channels, and blockade of Nav1.5 is considered as a safety risk for drug development. From SyncroPatch, V½ act./V½ inact. and k act./k inact. were -50.3 ± 0.5/-72 ± 0.4 mV and 2.7 ± 1.0/5.9 ± 0.4 mV/e-fold potential change, similar to -50.1 ± 0.4/-73.5 ± 0.7 mV and 3.5 ± 0.5/5.8 ± 0.6 mV/e-fold potential change from manual patch clamp with no statistically significant difference (Fig 1E and 1F), and also similar to literature [22]. Therefore SyncroPatch could recapitulate biophysical properties of Nav1.7 and Nav 1.5 channels.

Next, we characterized Kv1.3, a voltage-gated potassium channel. The V½ act./V½ inact. and k act./k inact. were -27.9 ± 1.0/-44.5 ± 0.5 mV and 8.5 ± 0.4/4.1 ± 0.4 mV/e-fold potential change from SyncroPatch, consistent with -29.4 ± 1.6/-45.2 ± 0.5 mV and 7.9 ± 1.4/4.2 ± 0.4 mV/e-fold potential change from manual patch, with no statistically significant difference (Fig 1G and 1H), and similar to literature [23].

### Nav1.7 APC parameter optimization

While SyncroPatch is capable of replicating biophysical parameters for Nav1.7, Nav1.5 and Kv1.3 channels, its application to drug screening requires more rigorous test. For quality control of data, we set up several parameters, including cell catching seal resistance (R_CATCH_), whole cell configuration seal resistance (R_SEAL_) and R_SEAL_ stability and baseline peak current amplitude (Table 1). After addition of cells and application of negative pressure, cell catching occurred as manifested by increasing R_CATCH_, which we set criterion 10 MΩ for cell catch related parameters optimization. Aiming for high quality recording, we set whole experiment R_SEAL_ > 500 MΩ and baseline peak current amplitude >500 pA (elicited by -10 mV pulse). Additional quality control (e.g., current rundown) was monitored by visual inspection.

**Table 1:** Acceptance criteria for automated patch clamp.

Parameter	Acceptance criterion
R_CATCH_ (cell catching)	> 10 MΩ
R_SEAL_ (experiment beginning)	> 500 MΩ
R_SEAL_ stability (experiment end)	> 500 MΩ
Current amplitude (experiment beginning)	> 500 pA

**Fig 2:**
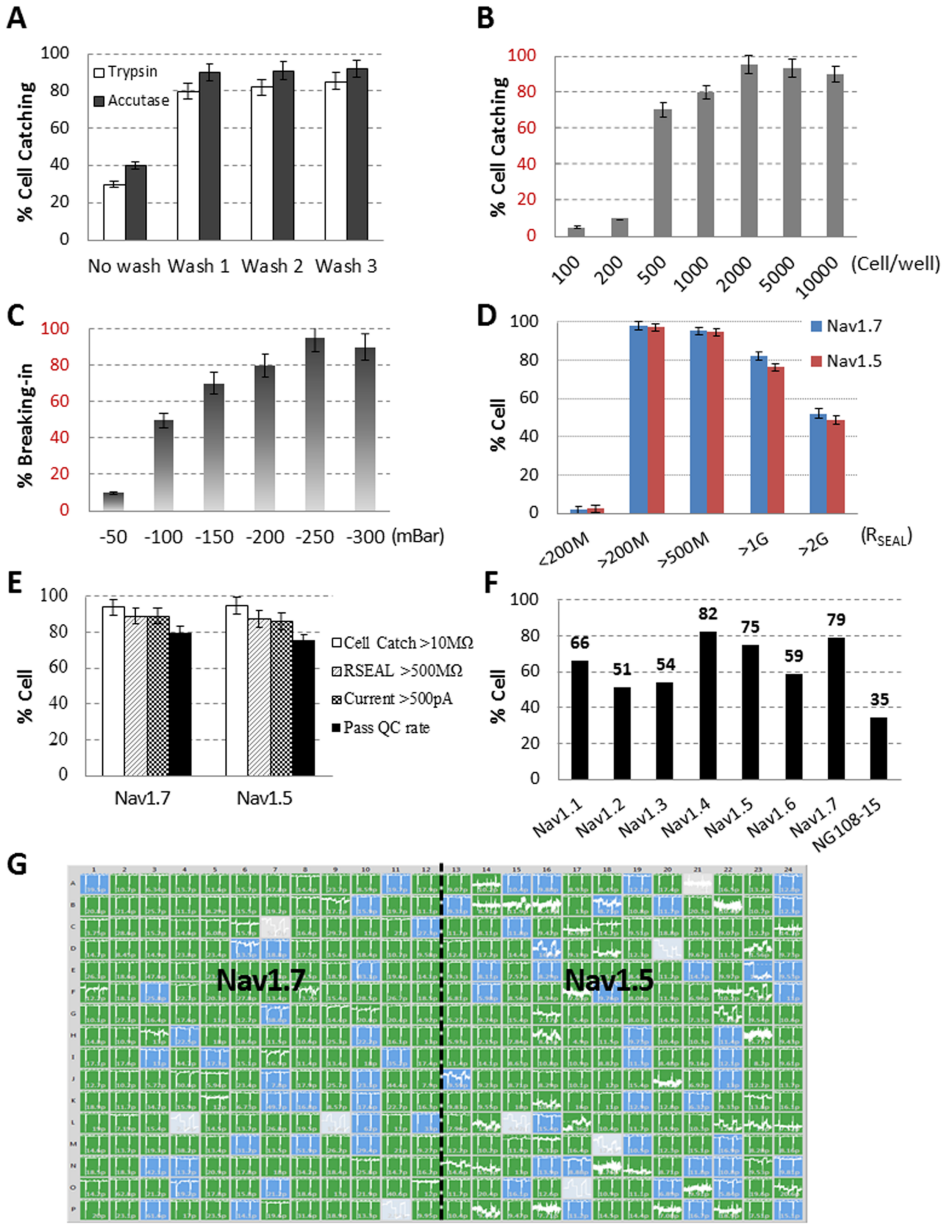
SyncroPatch cell patching success rate optimization. (A) Cell harvesting with accutase brought better cell catching rate than Trypsin, and 1 time cell washing with PBS significantly improved cell catching rate; (B) Increasing Cell number up to 2000 per well reached the best cell catching rate; (C) The best cell membrane breaking-in pressure for our tested Nav1.7 and Nav1.5 cell lines were at -250 mBar; (D) The distribution of R_SEAL_ from optimized Nav1.7 and Nav1.5 recordings; (E) Under optimized cell patching parameters, SyncroPatch CHO-Nav1.7 and CHL-Nav1.5 cell patching success rate by each criterion and all criteria. Note that all comparison experiments were done by fixing other parameters at the optimized condition and varying the experimental parameter only; (F) Under optimized APC parameters, voltage gated sodium current recording success rate from Nav1.1, Nav1.2, Nav1.3, Nav1.4, Nav1.5, Nav1.6, Nav1.7 and NG108-15 cell line was 66%, 51%, 54%, 82%, 75%, 59%, 79% and 35%, respectively; (G) A representative SyncroPatch recording of CHO-Nav1.7 (left half chip) and CHL-Nav1.5 (right half chip) in a 384 well chip. The R_SEAL_ in each well was indicated as less than 200 MΩ in gray, between 200 MΩ and 1 GΩ in blue, and bigger than 1 GΩ in green by using SyncroPatch PatchControl software; All data shown as mean ± SD, with data points in SyncroPatch n = 200~384.

We found cell catching was influenced by multiple factors, such as cell preparation, cell density and pressures for catching, sealing and rupturing of the cell membrane. Among the various cell preparation methods, we found that cell dissociation with accutase, coupled with one or more washing steps with PBS, produced good cell catching for the tested cell lines (Fig 2A, Nav1.7 as example). A possible explanation could be that accutase treatment may maintain membrane healthiness, and the washing step may reduce membrane debris which may interfere with cell catching. We also found that increasing cell number up to 2,000 cells per well and increasing cell membrane breaking-in pressure up to 250 mBar produced the best results (Fig 2B and 2C). The same protocols were also applicable to Nav1.5 and Kv1.3 cell lines. Fig 2G was a representative recording of Nav1.7 and 1.5 channels from a single chip plate, with R_SEAL_ color-coded (gray: < 200 MΩ; blue: 200- 1GΩ in blue, green: > 1GΩ). Based on baseline recording, 82.8% of Nav1.7 and 76.6% of Nav1.5 cells achieved giga-seal (> 1GΩ), 95.3% of Nav1.7 and 94.8% of Nav1.5 cells reached seal resistance > 500 MΩ (Fig 2D and 2G). Combining criteria of cell catching (> 10 MΩ), seal resistance (>500 MΩ) and baseline current amplitude (>500 pA), the overall success rate was 79% for Nav1.7 and 75% for Nav1.5. (Fig 2E). To test whether this optimized method will be applicable to a wide range of other voltage gated sodium channels, we performed a cross-comparison of 8 Nav channels, including 5 home-developed cell lines: Nav1.1, Nav1.2, Nav1.5, Nav1.6 and Nav1.7; 2 commercial available cell lines: Nav1.3 and Nav1.4; and neuroblastoma NG108-15 cells. Among 8 cell lines, 3 had success rates > 70%: Nav1.4 (82%), Nav1.7 (79%) and Nav1.5 (75%); 4 had success rates between 70~50%: Nav1.1 (66%), Nav1.6 (59%), Nav1.3 (54%) and Nav1.2 (51%); and neuroblastoma NG108-15 cells had success rate 35% (Fig 2F). This data suggested that this optimized method can achieve satisfactory results for most Nav expression cell lines, albeit further optimization may be necessary for some special cells such as neuroblastoma NG108-15.

**Fig 3:**
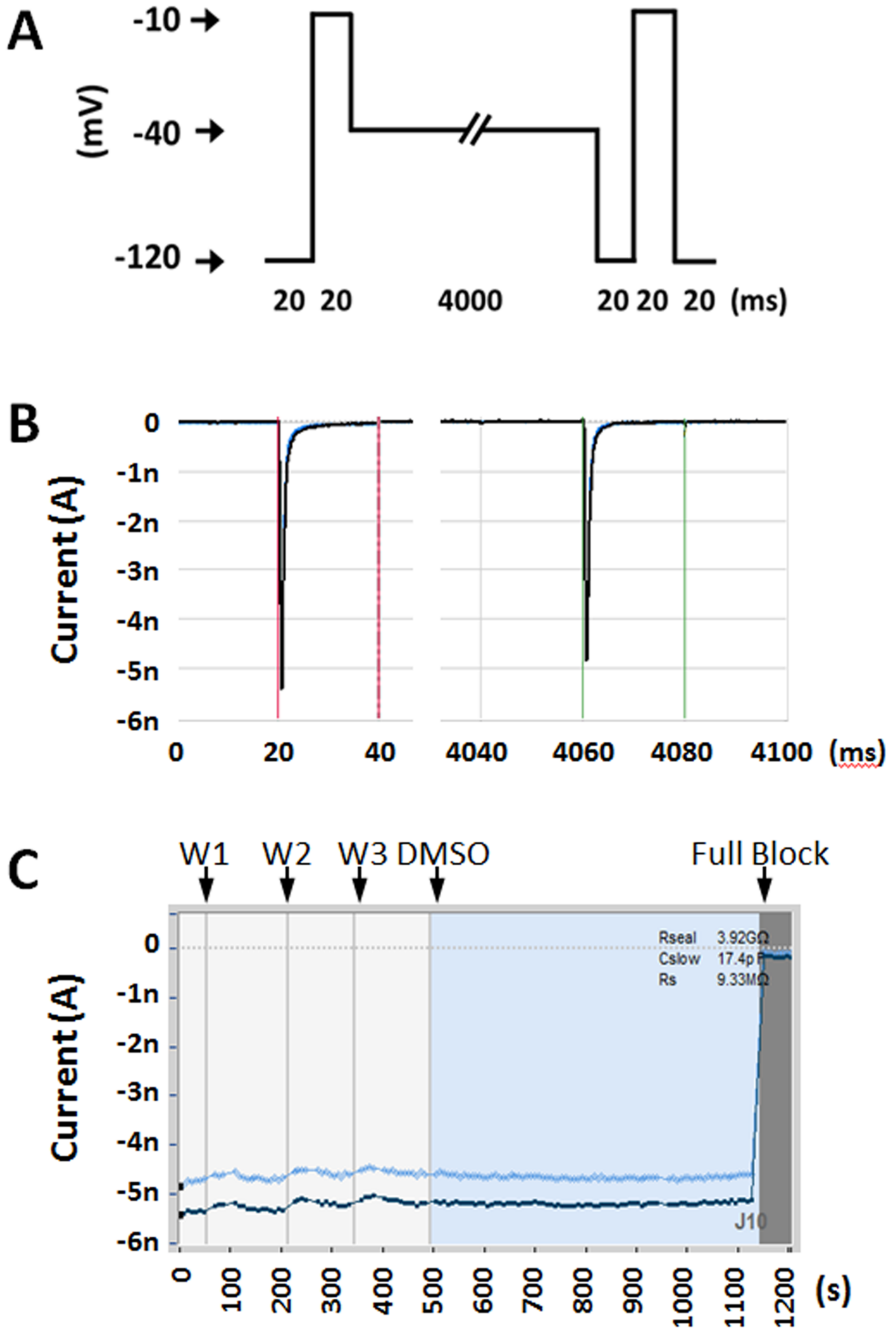
SyncroPatch Nav1.7 recording from optimized conditions with two-state voltage protocol. (A) Voltage protocol to elicit Nav1.7 currents from closed (-120 mV) and inactivated (-40 mV) states. (B) Representative single sweep Nav1.7 current under described voltage protocol. The closed and inactivated state elicited peak currents were auto selected between red and green cursors. (C) The same representative recording current time plot shows steady closed state (dark blue) and inactivated state (light blue) peak currents through the whole experiment, including 3 times external solution washing (W1, W2 and W3) and 10 mins after applying 0.2% DMSO external solution.

One frequent problem, with quantitative pharmacology study is current instability, which could be caused by deterioration of patch clamp parameters, or changing channel properties (e.g., slow inactivation). In manual recording of Nav1.7 >10% rundown is often observed within the first 15 min of recording. Initially, we observed > 30% rundown on the SyncroPatch. To mitigate this issue, we optimized several parameters, including temperature of the cell hotel (10°C), external and internal solutions compositions (see Methods). We devised a voltage protocol (as shown in Fig 3A), which could be used to assess both closed state and inactivated state Nav1.7 channel block. Under this protocol, the peak currents (V_open_ at -10 mV pulses) from both closed (-120 mV) and inactivated (-40 mV) states showed <5% rundown during 20 minutes’ recording (Fig 3B and 3C). Even though this protocol might not be ideal for testing compounds with very slow kinetics and exclusively one state dependent binding, it is suitable as a front line assay.

### Test of reference Nav1.7 inhibitors

**Fig 4:**
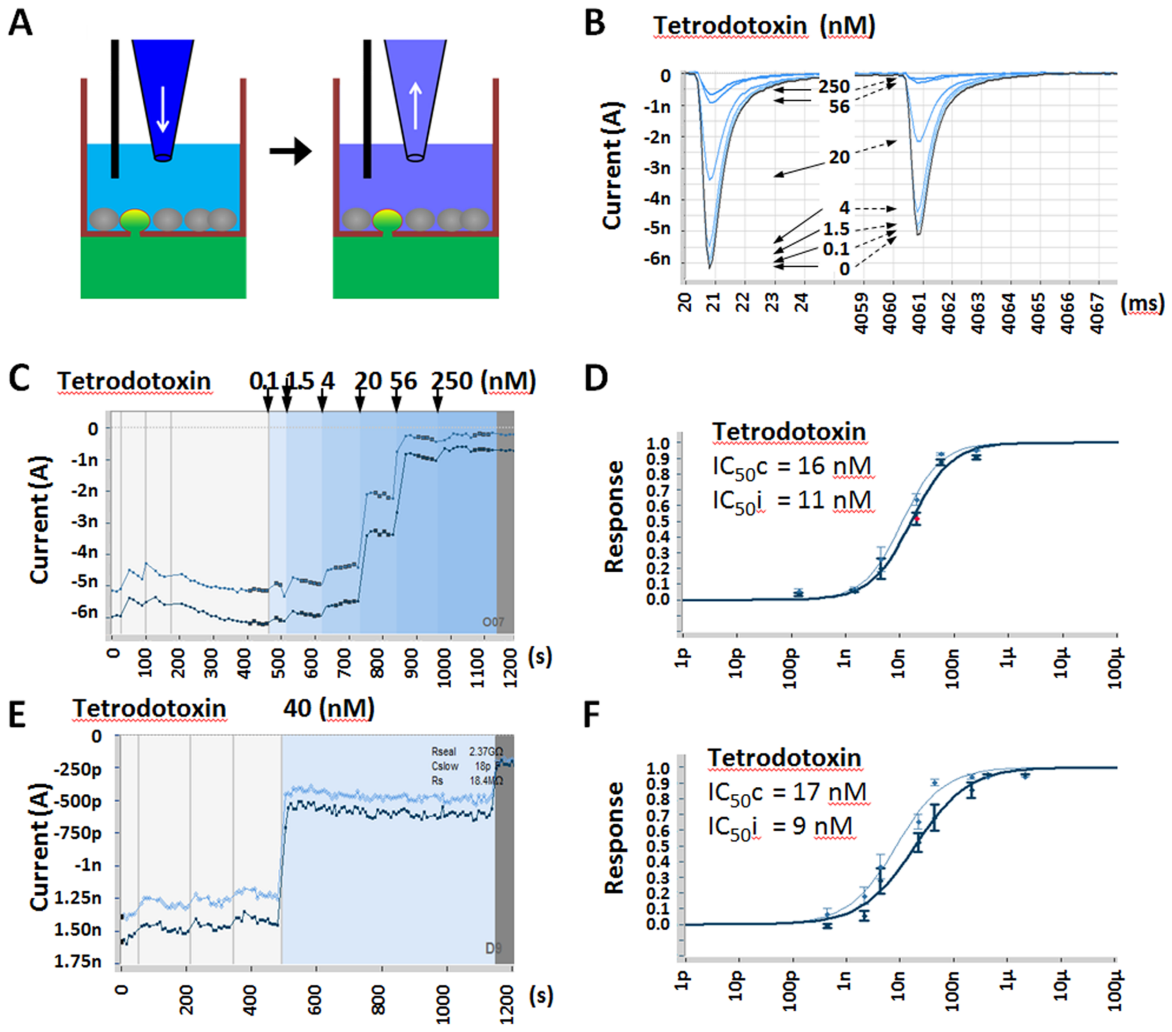
SyncroPatch hNav1.7 dose response study by using multiple and single concentration per well methods. (A) Chamber configuration and compound solution addition and removal to achieve increasing compound concentrations in a single well. (B) Representative Nav1.7 current traces from closed and inactivated states at before (highlighted) and after sequential increasing Tetrodotoxin (TTX) concentrations from 0, 0.1, 1.5, 4, 20, 56 to 250 nM. (C) The same representative recording peak current time plot shows Nav1.7 closed state (dark blue) and inactivated state (light blue) inhibition by increasing concentration of TTX, with TTX concentration changes indicated by background colors. (D) TTX dose–response curves for Nav1.7 closed state (dark blue) and inactivated state (light blue) using 6 concentrations per well protocol. (E) (F) Same experiment as (C) (D) by using single dose per well protocol.

We next tested this optimized protocol with a set of reference compounds. Unlike manual patch clamp or Qube, which uses a flow through design, SyncroPatch uses a Biomek liquid pipetter to add and aspirate solution (Fig 4A). The question arises as to whether SyncroPatch could test multiple concentration compound effects on a single cell. To this end, we tested Tetrodotoxin (TTX), a fast onset compound, by comparing multiple vs. single concentration methods. For multiple concentrations, we sequentially added and aspirated ascending concentrations of TTX (0.1, 1.5, 4, 20, 56 and 250 nM) to single cell (Fig 4B). TTX IC_50_s were determined to be 16 nM at closed state and 11 nM at inactivated state (Fig 4D). These values were consistent with IC_50_s obtained from single concentration method, 17 nM at closed and 9 nM at inactivated states (Fig 4E and 4F), and were also similar to literature reports [24–26]. Therefore we proved that multiple data points can be generated form a single cell in SyncroPatch APC system. Despite a much lower overall experimental success rate of 36%, compared to the single concentration method of 79%, this multiple concentration method still has advantages in fully utilizing SyncroPatch flexibility and further boosting APC assay throughput.

**Fig 5:**
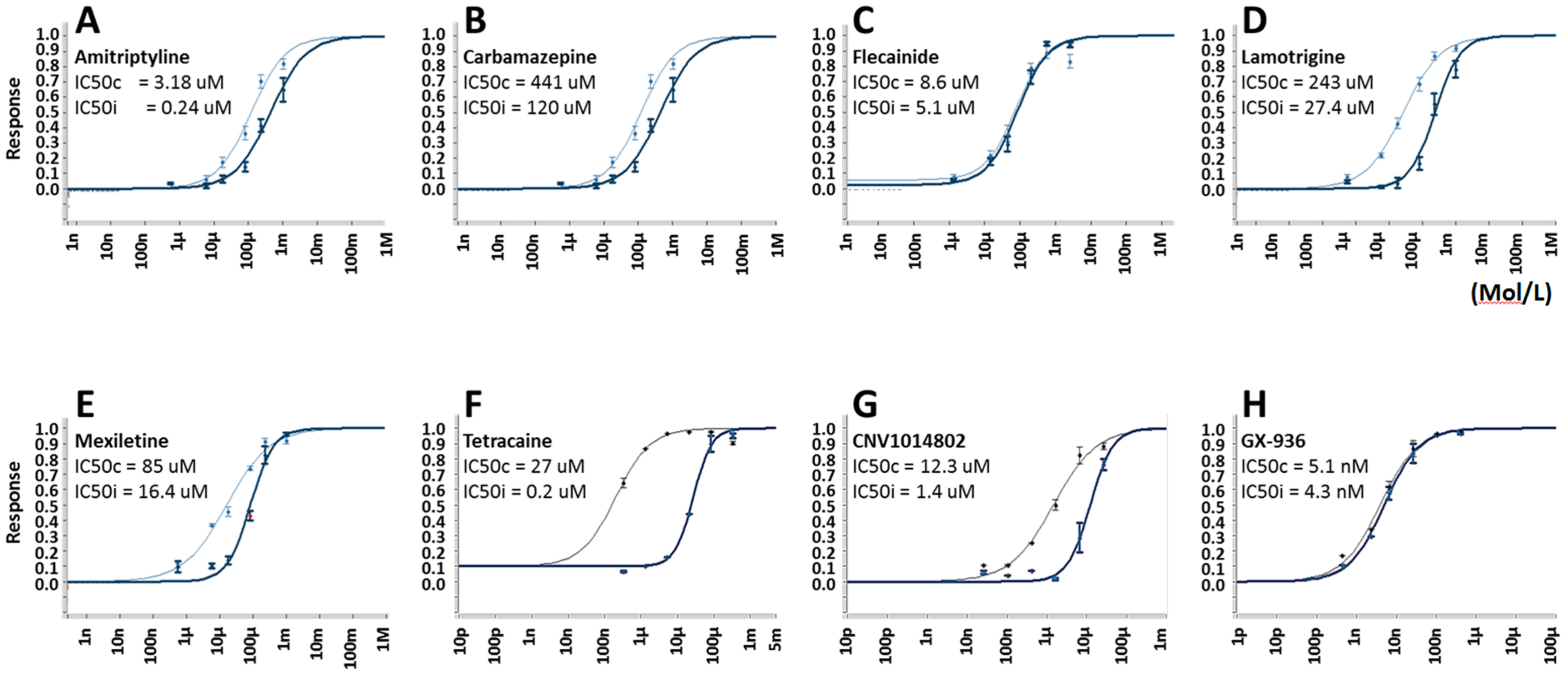
Dose response of reference sodium channel blockers on hNav1.7. Data were obtained using SyncroPatch multiple concentration per well method. (A-H) Amitriptyline, Carbamazepine, Flecainide, Lamotrigine, Mexiletine, Tetracaine, CNV1014802 and GX-936 respectively. IC_50_ at closed state (IC_50_c, bold line) and inactivated state (IC_50_i, thin line) were calculated by fitting to four-parameter Hill equation; all data shown as mean (n = 12~24).

Using this multiple concentration from a single cell method, we further determined IC_50_s from additional reference compounds, including pore blockers (Amitriptyline, Carbamazepine, Flecainide, Lamotrigine, Mexiletine, Tetracaine, CNV1014802 [26]) and a recently reported VSD4 blocker GX-936 [27]. For each compound, inhibition from closed state and inactivated state was determined (Fig 5A–5H). CNV1014802 and Tetracaine exhibited strong state dependence, with over 10 fold difference in potency between closed and inactivated states; Amitriptyline, Carbamazepine, Lamotrigine, Mexiletine showed less pronounced state dependency, whereas Tetrodotoxin, Flecainide and GX-936 did not show state dependency (Table 2). These data from our developed SyncroPatch APC assay are largely consistent with literature reports (Table 2).

**Table 2:** Comparison of reference compounds IC_50_ (uM) values generated from single and multiple concentrations methods and literature reports.

Compounds	Single Dose Closed State	Six Dose Closed State	Literature Closed State	Single Dose Inactivated state	Six Dose Inactivated state	Literature Inactivated state	Reference
Tetrodotoxin	0.017	0.016	0.02	0.009	0.011	0.02~0.03	[20],[19]
Amitriptyline	3.74	3.18	10.01	0.147	0.245	0.56~1	[16],[19]
Carbamazepine	497.4	441	>100	88.4	120	29~101	[19],[20]
Flecainide	6.69	8.6	>10	2.69	5.1	0.85	[16]
Lamotrigine	311.9	243	>100	16.5	27.4	34~79	[19],[20]
Mexiletine	117.7	85	121.94	15.3	16.4	30.36~47	[19]
Tetracaine	20.7	27.0	20~30	0.264	0.245	0.1~0.43	[16],[19],[20]
CNV1014802	46.7	12.3	54	1.41	1.4	6.3	[21]
GX-936	0.007	0.0051		0.003	0.0043		

### Pilot screen of 10,000 compounds

**Fig 6:**
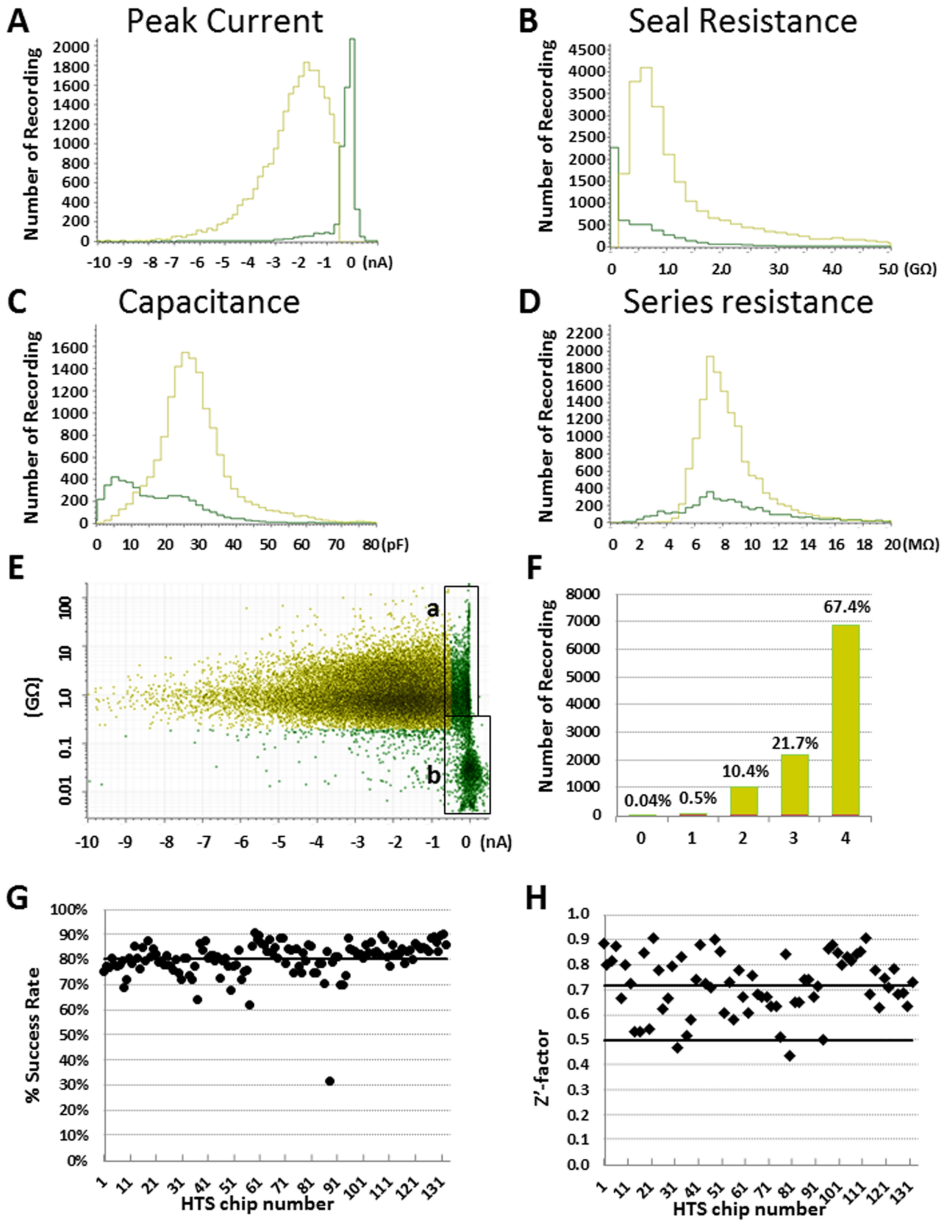
Quality analysis of 10,000 compounds Nav1.7 pilot screening. Total 42,272 data points were analyzed, including n = 4 for each compound and 0.2% DMSO negative controls. Each parameter’s binned mean values were plotted with frequency N numbers in histograms for (A) peak current, (B) seal resistance, (C) capacitance, (D) series resistance. In each graph, yellow color line represents QC passed tests, and green color line represents QC failed tests by all criteria; (E) Scatter plot of baseline signal and seal resistance of each recording. Green color data points are QC failed recordings from all criteria. Box (a) shows data failed by peak current criterion due to low Nav1.7 expression. Box (b) shows data failed by both peak current and seal resistance criteria. (F) Histogram of compounds with 0 to 4 valid data points from 4 repeats. The percentage of having 4, 3, 2, 1, and 0 QC passed recordings were 67.4%, 21.7%, 10.4%, 0.5% and 0.04%, respectively. (G) Scatter plot of percentage success rate for each chip, and the median success rate 79% is indicated with a solid line. (H) Scatter plot of Z’-factor for each chip with DMSO controls. The median Z’-factor is indicated by a solid black line at 0.72.

To test SyncroPatch in a drug screening setting, we did a pilot screen of 10,000 compounds contained in 32 384-well plates and each plate was tested 4 times. The final testing concentration was at 6.7 μM. The whole screening was finished in 8 days with daily throughput ~6,000 data points by running 16 test chips. To assess assay performance from all 42,272 test points, four parameters were determined: the average baseline peak current which indicates current density, seal resistance, cell capacitance and series resistance. The average baseline peak current at closed state was 1.66 ± 0.01 nA, and 82% recordings had currents > 0.5 nA (Fig 6A); the remaining 18% had current less than -0.5 nA due to low Nav1.7 expression or failure in forming whole-cell configuration (Fig 6E, Box a & b). The average seal resistance was 0.95 ± 0.05 GΩ, and seal resistance > 2, 1, 0.5 and 0.2 GΩ recording rates were 30%, 62%, 90% and 92%, respectively (Fig 6B). The average cell capacitance was 25 ± 0.1 pF (n = 42,272); and 95% wells had capacitance between 10 to 40 pF (Fig 6C). The average series resistance was 7.9 ± 0.1 MΩ, and 95% recordings had series resistance between 5 to 12 MΩ (Fig 6D). By testing each compound with n = 4 repeats, 67.4% compounds had 4 valid data points, 21.7% had 3 data points, 10.4% had 2 data points, 0.5% have one data point; and only 0.04% compounds failed (Fig 6F). Therefore for the 10,000 compounds screening, the compound testing success rate was 99.96%. For each 384 well plate, the median success rate was 79% and was maintained steady through the screen. The high screening quality was also reflected by median Z’-factor 0.72 [28]. 98.5% plates showed Z’-factors > 0.5, with the other two Z’-factors at 0.43 and 0.47 (Fig 6H). Overall these data suggested that our developed Nav1.7 SyncroPatch APC assay was robust and suitable for high-throughput screening.

### Recording Kv1.3 currents from primary T cells

**Fig 7:**
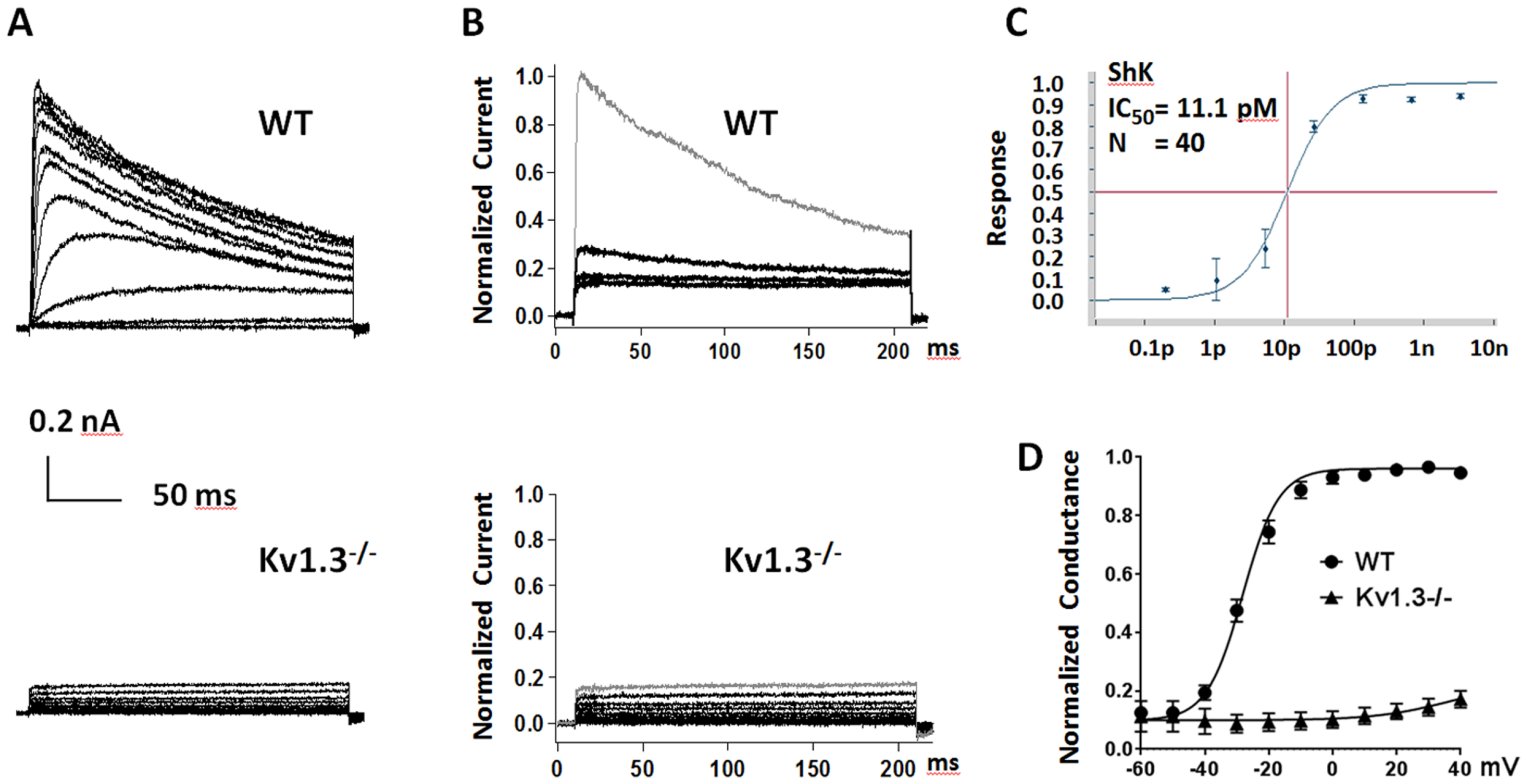
SyncroPatch APC endogenous voltage-gated potassium channel Kv1.3 from primary T cells. (A) Representative Kv1.3 currents from WT and *Kcna3*^-/-^ T cells. Currents were elicited by depolarizing voltage steps from −60 mV to +40 mV (10 mV increments, with −80 mV membrane holding potential) every 30 seconds; (B) Normalized WT and *Kcna3*^-/-^ T cell K^+^ currents from before (gray) and after Shk 1nM inhibition (black). 89% WT T cell K^+^ current was blocked by Shk 1nM, and no Shk sensitive current was detected in *Kcna3*^-/-^ T cells; (C) Dose response curve for Kv1.3 inhibition by ShK, with IC_50_ = 11.1pM. Data was generated using SyncroPatch APC platform with n = 40; (D) The conductance-voltage curves from WT and *Kcna3*^-/-^ T cells were fitted with Boltzmann function and the V½ act. -28.1±1 mV was detected from WT T cells. Data are presented as the means ± SEM, with WT T cells (black dot) n = 40 and *Kcna3*^-/-^ T cells (black triangle) n = 50.

Direct recording from primary T cells present a significant challenge, partially due to its small size (~7 μm diameter after activation), irregular shape and fragile membrane. Therefore we explored whether SyncroPatch could be utilized here. We tested various parameters for cell catching, cell membrane breaking-in and different chips with varying cell catching hole sizes (represented by chip resistance from 3 to 10 MΩ). We found that two 1,000 ms suction pulses of 200 mBar and high resistance chip (10 MΩ) produced the best results. Under these conditions, 11.7% of recordings met QC criteria (Table 1). Theoretically, the success rate could be further improved by reducing the chip hole size (resistance > 10 MΩ). As a test case, we characterized Kv1.3 currents in T cells isolated from WT and *Kcna3*^-/-^ (knockout) rats (Fig 7A). To distinguish Kv1.3 activity from leak and other potassium channel currents, we used the specific Kv1.3 blocker, ShK at 1 nM, ~100 times of its IC_50_ 11 pM (Fig 7B and 7C). ShK sensitive potassium current was obtained for conductance calculation (Fig 7D). The V_1/2_ activation from WT primary T cells was -28.1±1 mV, similar to exogenously expressed Kv1.3 in CHO cells (29.4±1.6 mV, Fig 1G and 1H). Additionally, no Kv1.3 activity was detected from *Kcna3* ^-/-^ T cells. Therefore Kv1.3 encodes the dominate potassium currents in T cells and SyncroPatch was proven feasible in primary T cell recording.

## Discussion

Besides SyncroPatch, another two comparable APC systems, IonWorks Barracuda and Qube, each with a single 384 module, also have the capacity of parallel recording of 384 cells. IonWorks Barracuda was launched in 2010 and has been reported for compound screening on hERG, Ca_V_2.2 and Nav channels [16–18]. Different from the other two systems, Barracuda uses perforated patch configuration, and the averaged seal resistance was ~120 MΩ for single-hole mode and ~35 MΩ for population patch mode (64 holes). Qube was introduced in 2014 and shared many similar features with SyncroPatch, including using a 384 format design (e.g., 384 channel digital amplifier, 384 pipetting robot and 384 well borosilicate glass chips) and a programmable negative pressure system to achieve whole cell configuration, thus with potential for giga-seal quality recording. In a recently reported Nav1.7 modulator screening by using Qube system, ~80% wells achieved > 15 MΩ seal resistance (in 10-hole population patch mode), equating to 150 MΩ resistance for each single cell recording, with daily throughput ~2300 compounds [29]. SyncroPatch 768PE was also developed in 2014 using negative pressure to form high quality whole cell configuration as Qube, but was equipped with different amplifier, non-micro-fluid planar chip design, and two-384 modules on a Biomek automation workstation. In our optimized Nav1.7 inhibitor screening using SyncroPatch 768PE system, 92% recordings achieved > 200 MΩ seal resistance, and 90% recordings reached > 500 MΩ seal resistance (Fig 6B), and the daily throughput was ~6,000 compounds. Our data suggested that SyncroPatch 768PE can generate both higher-quality and higher throughput data in our optimized Nav1.7 assay.

Unlike Qube which utilizes a flow through design, SyncroPatch uses liquid handler (BioMek) to add and aspirate solution. The question arises as to whether SyncroPatch could achieve sufficient solution switch, and could be used to test multiple doses of compounds on a single cell. Therefore we developed a protocol by using Biomek to add and aspirate solutions, so ascending compound concentrations (up to 6 in total) could be tested in a single cell. We demonstrated that potency of reference compounds were consistent between the multi-dose protocol and single concentration protocol, and were consistent with literature data. Note that, due to repeated solution addition and aspiration in the multi-dose protocol, the whole experiments success rate was only ~36%. Thus multi-dose protocol should only be used when throughput is a higher priority than success rate. In our pilot screen using the single dose method, we demonstrated the assay was highly robust with daily throughput ~6,000 data points and Z’-factor 0.72. Benefiting from the SyncroPatch’s inbuilt liquid handling robot, its future throughput can be further increased by implementing solution auto feeding system and plate stacker to develop unattended operation for long period auto screening.

Another major consideration in designing an appropriate APC HTS assay would be data reduction and HTS data analysis. We focused on four key quality control parameters, seal resistance, baseline peak current amplitude, cell capacitance and series resistance to optimize our assay QC criteria. By comparing each parameter’s distribution and median value, we set cutoff at seal resistance 0.5 GΩ and peak current 0.5 nA as QC criteria to exclude recording data from low quality cell patching and good cell patching but low channel expression. These criteria were validated using reference Nav1.7 inhibitors and applied to the pilot 10,000 compound screening. As the nature of APC assay is a single cell based assay, for each APC screening campaign requiring high volume data analysis and rapid turnaround, pilot reference compound studies, that allow for further optimization of assay protocol and QC criteria for the classes of compounds to be tested, will be necessary.

For the main application of using transgenic stable cell lines for target ion channel study, it will be of great benefit to selected a host cell line with good membrane electrophysiological properties, such as homogeneous high level expression of target ion channel for robust recording signal, big cell capacitance for efficient cell catching, good membrane property for easier cell breaking-in to make whole cell confirmation and longtime stable recording. In this study we screened 48 individual clones in each cell line development, and optimized APC parameters which proved can achieve satisfactory results for most Nav expression cell lines with recording success rates ranging between 51% ~ 82%. The relatively low success rate 35% from neuroblastoma NG108-15 cell recording can be caused by two reasons: 1) the cell was recorded in undifferentiated condition with low Na^+^ currents density -34.2 pA/pF (S1 Fig), similar to literature data [30]; 2) the cell catching hole size was not optimized for this special cell line. In this study, we used customized medium resistance (5~8 MΩ) chip for all stable cell lines study. Therefore, other than those described parameters, special cell line APC recording optimization should also focus on increasing target expression level and chip customization. Overall judging by criteria in Table 1, our final optimized APC assay success rate for Nav1.7 was 79%, which was sufficient for high throughput study.

Primary cell recording is another important area for ion channel research but remains a significant challenge. To date, all APC systems have been mainly focused on studying ion channel using stable cell lines, except one report using Patchliner, an 8 cell parallel recording system, to record currents from several primary cells [31]. The success rate ranged from 8.3% to 60%, with T-lymphoblast being particularly difficult. Here we showed that by optimizing SyncroPatch 768PE system we could obtain T cell recording success rate 11.7% in 768 parallel recording format. Thus, SyncroPatch provides a feasible platform for studying endogenous ion channels in primary T cells.

In summary, we developed a robust high-throughput electrophysiological assay for Nav1.7 by using the newest generation APC system SyncroPatch 768PE. Our data suggested that this system is able to produce giga-seal quality recording data with daily throughput ~6,000 data points and Z’-factor 0.72, and also can be used for primary T cell recoding. Thus, the voltage-gated ion channels’ study is reinforced by SyncroPatch 768PE and will have a significant impact on ion channel research and the new generation of ion channel target drug discovery and development.

## Supporting information

S1 Fig Voltage-gated Na^+^ current in undifferentiated neuroblastoma NG108-15 cells. (A) Na^+^ current density was calculated by using peak current elicited by 20 ms test pulses from -100 to 0 mV, and divided by cell capacitance. The median Na^+^ currents density was -34.2 pA/pF; (B) Na^+^ current characterization by steady-state activation and inactivation curves. The smooth curves are Boltzmann fits, and the half-activation/inactivation voltages (V½ act./V½ inact.) and slope factors (k act./k inact.) are -19.0 ± 0.1/-65.7 ± 0.1 mV and 4.5 ± 0.1/6.8 ± 0.1 mV from APC. Note that all data were shown as mean ± SEM, with data points in SyncroPatch APC n = 266.
(TIF)
