# Supplementary figures and images for: High-throughput electrophysiological assays for voltage gated ion channels using SyncroPatch 768PE
Source: PLoS One. 2017 Jul 6;12(7):e0180154. doi: 10.1371/journal.pone.0180154 (PMC5500279; doi:10.1371/journal.pone.0180154)

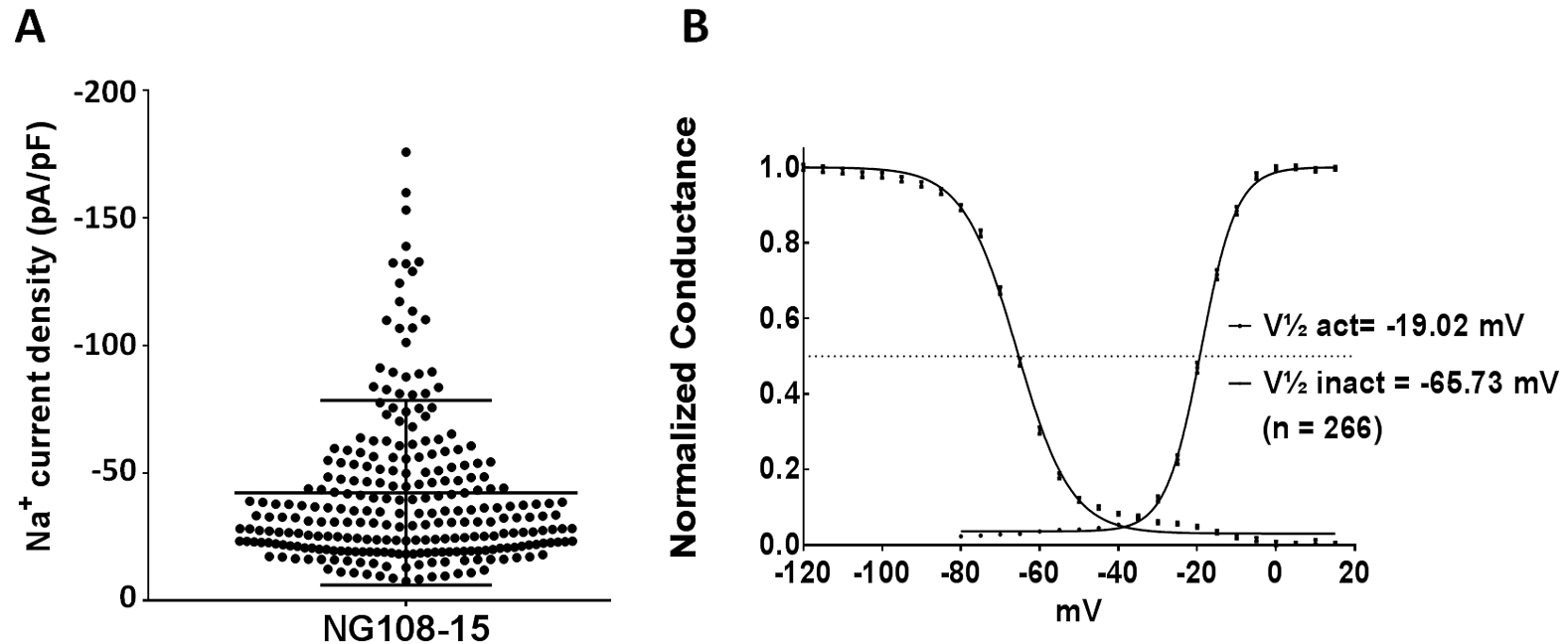

Supplement: S1 Fig — (A) Na+ current density was calculated by using peak current elicited by 20 ms test pulses from -100 to 0 mV, and divided by cell capacitance. The median Na+ currents density was -34.2 pA/pF; (B) Na+ current characterization by steady-state activation and inactivation curves. The smooth curves are Boltzmann fits, and the half-activation/inactivation voltages (V½ act./V½ inact.) and slope factors (k act./k inact.) are -19.0 ± 0.1/-65.7 ± 0.1 mV and 4.5 ± 0.1/6.8 ± 0.1 mV from APC. Note that all data were shown as mean ± SEM, with data points in SyncroPatch APC n = 266. (TIF) [file pone.0180154.s001.tif]
